# High-resolution and accelerated multi-parametric mapping with automated characterization of vessel disease using intravascular MRI

**DOI:** 10.1186/s12968-017-0399-6

**Published:** 2017-11-20

**Authors:** Guan Wang, Yi Zhang, Shashank Sathyanarayana Hegde, Paul A. Bottomley

**Affiliations:** 1 Department of Electrical & Computer Engineering, Johns Hopkins University, Baltimore, MD USA; 2 Division of MR Research, Department of Radiology and Radiological Sciences, Johns Hopkins University, Park building 310, 600 N Wolfe Street, Baltimore, MD 21287 USA

**Keywords:** Intravascular MRI, Atherosclerosis, Relaxation times, Disease classification, Accelerated acquisition, SLAM, Machine learning

## Abstract

**Background:**

Atherosclerosis is prevalent in cardiovascular disease, but present imaging modalities have limited capabilities for characterizing lesion stage, progression and response to intervention. This study tests whether intravascular magnetic resonance imaging (IVMRI) measures of relaxation times (T_1_, T_2_) and proton density (PD) in a clinical 3 Tesla scanner could characterize vessel disease, and evaluates a practical strategy for accelerated quantification.

**Methods:**

IVMRI was performed in fresh human artery segments and swine vessels in vivo, using fast multi-parametric sequences, 1–2 mm diameter loopless antennae and 200–300 μm resolution. T_1_, T_2_ and PD data were used to train a machine learning classifier (support vector machine, SVM) to automatically classify normal vessel, and early or advanced disease, using histology for validation. Disease identification using the SVM was tested with receiver operating characteristic curves. To expedite acquisition of T_1_, T_2_ and PD data for vessel characterization, the linear algebraic method (‘SLAM’) was modified to accommodate the antenna’s highly-nonuniform sensitivity, and used to provide average T_1_, T_2_ and PD measurements from compartments of normal and pathological tissue segmented from high-resolution images at acceleration factors of *R* ≤ 18-fold. The results were validated using compartment-average measures derived from the high-resolution scans.

**Results:**

The SVM accurately classified ~80% of samples into the three disease classes. The ‘area-under-the-curve’ was 0.96 for detecting disease in 248 samples, with T_1_ providing the best discrimination. SLAM T_1_, T_2_ and PD measures for *R* ≤ 10 were indistinguishable from the true means of segmented tissue compartments.

**Conclusion:**

High-resolution IVMRI measures of T_1_, T_2_ and PD with a trained SVM can automatically classify normal, early and advanced atherosclerosis with high sensitivity and specificity. Replacing relaxometric MRI with SLAM yields good estimates of T_1_, T_2_ and PD an order-of-magnitude faster to facilitate IVMRI-based characterization of vessel disease.

**Electronic supplementary material:**

The online version of this article (doi:10.1186/s12968-017-0399-6) contains supplementary material, which is available to authorized users.

## Background

Atherosclerosis is the prevalent factor in patients presenting with cardiovascular disease, which is responsible for ~1/3 of all deaths in the United States and includes heart disease, stroke, peripheral artery disease, and hypertension [1]. Because plaque evolution from early-stage lesions to those vulnerable to rupture can take decades, differentiation of lesion stage is crucial for assessing progression and measuring the efficacy of dietary, pharmaceutical and intravascular interventions. The American Heart Association (AHA) has classified atherosclerotic lesions as Types I-VI based on the presence of foam cells; extra-cellular lipids; fibrous caps that have a high risk of rupture and causing stroke or myocardial infarction; calcification; fibrosis, and complex ruptured thrombotic lesions [2].

Clinically, atherosclerosis is diagnosed by the presence of significant luminal narrowing using X-ray angiography [1]. However, because X-ray cannot detect vessel wall, non-calciferous lesion morphology or early-stage lesions with mild stenosis [3], it is insensitive to most of the AHA metrics that define disease stage. While both intravascular ultrasound (IVUS) and optical coherence tomography (OCT) *can* provide transluminal imaging [4], they also require X-ray guidance and may be confounded by calcification [5, 6], or penetration depth and the need for a blood-free environment [4], respectively. Advances in all of these modalities are promising [7], but no single technique is presently suited for minimally-invasive assessment of all of the relevant AHA disease characteristics needed to monitor plaque progression or regression [8] or to document the efficacy of therapy or lifestyle changes.

Although noninvasive angiography of vessel lumens with magnetic resonance imaging (MRI) has only modestly impacted vessel wall imaging, MRI’s intrinsic sensitivity to soft tissue in noninvasive spin-lattice (T_1_) and spin-spin (T_2_) relaxation-weighted ‘black blood’ studies has permitted differentiation of healthy vessel from atherosclerosis [9–11]. Indeed, meta-analysis of studies employing combined relaxation, proton density (PD) and/or flow weightings shows that MRI detection of intra-plaque hemorrhage in carotid disease is highly predictive of cerebrovascular events [12]. Nevertheless, with a typical carotid artery ≤0.75 mm thick [13] and MRI’s spatial resolution of ~0.4 mm [9, 10], vessel walls may occupy only 1-2 pixels. While spatial resolution and low signal-to-noise ratio (SNR) represent fundamental limits to accurate quantification, current *relaxation-weighted* MRI approaches for assessing vessel disease [11, 14, 15] can also confound multi-study comparisons and reproducibility for disease classification.

Unlike relaxation-weighted image intensities, the *absolute* T_1_, T_2_ and PD values reflect intrinsic properties of the biological tissue, and might provide more robust metrics for classifying vessel disease by ameliorating instrumental and sequence effects [11, 16]. Several studies have reported relaxation times for atherosclerotic tissues [17–22]. However, T_1_ and T_2_ measurements in vivo are also limited by long scan times and poor SNR at the spatial resolution needed to image thin vessel walls, which compromise quantification. Consequently, multi-parametric criteria for classifying vessel disease have yet to be developed.

Intravascular (IV) MRI coils [23–25], especially those operating at higher magnetic field strengths (*B*_*0*_ ≥ 3 Tesla, T) [25, 26], could dramatically improve spatial resolution and reduce scan times as a result of an intrinsic, realizable, *B*_*0*_^2^-dependence of their SNR [27, 28]. Biocompatible 3 T IVMRI coils have visualized calcification, pathology, accurately measured the thickness of fibrous caps on atherosclerotic plaques down to a native resolution of 80 μm in several minutes [29], and have performed ‘MRI endoscopy’ at 1-2 frames(fr)/s and 300 μm resolution [25]. Thus, IVMRI could provide the spatial resolution and SNR needed to characterize vessel disease. However, a practical and efficient strategy for acquiring the relaxometric information, would still be required. Unfortunately, the multi-coil based acceleration techniques used in conventional MRI (sensitivity encoding–‘SENSE’ and the like) are inapplicable to IVMRI which typically involves but a single detector.

Recently, the ‘SLAM’ (*spectroscopy with linear algebraic modeling*) localization method was developed to reduce scan-times for spectroscopy [30–33] and relaxometry [34], by directly encoding tissue ‘compartments’ using a small subset of the highest-SNR spatial-encoding steps. Acceleration factors of up to *R* = 120 have been demonstrated [30, 31]. The compartments are defined as areas of relatively uniform-appearing tissue on regular high-resolution or scout images. These can be segmented, re-segmented and reconstructed post-acquisition from the same undersampled data set, using high-resolution scout images as prior knowledge. SLAM could enable highly-accelerated compartment-average tissue characterization when the acquisition of high-resolution relaxation and/or PD maps is precluded by scan-time constraints [34]. Unfortunately, the standard SLAM method assumes that the detector phase and coil sensitivity are relatively uniform across segmented compartments [30, 31]. This is rarely true for IVMRI detectors, whose sensitivity is highly non-uniform [27, 28]. Thus, compensation for detector inhomogeneity would be key to accelerated IVMRI relaxometry using SLAM.

Here we posit that the measurement of T_1_, T_2_, and PD with high-resolution IVMRI could enable the characterization of atherosclerosis in situ. We further posit that SLAM can be adapted for IVMRI and used to provide accurate, highly-accelerated, measurements of compartment-average MRI relaxation times, and thereby enable the characterization of plaque components when high-resolution relaxometry is impractical due to scan-time limitations.

For the first time we present high-resolution IVMRI T_1_, T_2_, and PD results from autopsied human specimens and swine in vivo. The data were acquired using two fast MRI relaxometry methods: (i) the ‘four flip-angle’ (Four-FA) method, which uses the theoretical minimum number of steady-state acquisitions [35]; and (ii) the ‘mixed turbo spin-echo’ (MIX-TSE) method [36]. We then trained a supervised machine learning classifier—a support vector machine (SVM) [37, 38]—to automatically classify the stage of vessel disease from the multi-parametric data using histology as a standard. Finally, we adapted the SLAM method to provide highly-accelerated relaxometry with IVMRI detectors, by incorporating simple phase and amplitude sensitivity corrections into the reconstruction. SLAM was applied in vitro and in vivo to provide compartment-average T_1_, T_2_ and PD characterization up to 18-fold faster than the fully-sampled IVMRI acquisitions used for validation.

## Methods

### Subjects

Fresh human iliac and coronary artery segments (*n* = 10) were harvested from decedents (*n* = 6; age ≥ 60 yrs) in our Pathology Department with Institutional Review Board approval. MRI was performed shortly after harvest (<48 h). All decedents had a diagnosis of cardiovascular disease and atherosclerotic lesions were evident at gross examination in six specimens. Additional formalin-fixed specimens (excluded from the classifier training set) were used to test the robustness of the SVM algorithm. Specimens were 1-5 cm long, with 0.5-2.2 cm diameter lumens.

To test and compare relaxometry in vivo with the in vitro results in normal vessels, approval was obtained from our Institutional Animal Care and Use Committee for an in vivo study of a female Yorkshire pig. The animal was sedated with an intramuscular combination of ketamine, xylazine and telazol; induced intravenously with propofol to effect (approximately 4 mg/kg); and intubated and maintained with general anesthesia during IVMRI using 1%–2% isoflurane under mechanical ventilation. After MRI, the animal was humanely euthanized and vessel specimens harvested.

Transverse sections of the specimens were stained using hematoxylin and eosin (H&E; enhances calcification, lipid core and intra-plaque hemorrhage), *von Kossa* (enhances mineralization), *Verhoeff-van Gieson* (VVG; enhances elastic fibers) or *Movat* (enhances constituents of cardiovascular tissue) methods to visualize tissue histology.

### High resolution IVMRI

IVMRI was performed on a Philips 3 T Achieva clinical scanner (Philips Healthcare, Best, The Netherlands) using in-house loopless antenna IVMRI coils operating as receivers with body coil excitation. During IVMRI, RF heating of the antenna coils was controlled within 1 °C using decoupling circuitry and tuned connection cables, as verified by phantom studies and examination of in vitro and in vivo specimens described previously [28, 29].

In vitro studies of specimens were performed using a 2.2 mm outer diameter (OD), 400 mm-long semi-rigid copper coaxial cable antenna (UT-85-C, *Micro-coax Inc.*, Pottstown PA), with a distal whip formed by extending the central conductor by 39 mm [28, 29]. With the antenna located in the lumen, specimens were mounted at the center of a phantom filled with 3.5 g/l saline to mimic the body’s electrical properties at the MRI frequency, and scout MRI was performed. The Four-FA method was applied with a sequence repetition period T*R* = 651 ms; FAs of θ_1-4_ = 30°, 80°, 140°, 30°; and a three-dimensional (3D) pixel size =0.2 × 0.2 × 1.6mm^3^, to acquire four signals, S_1-4_ [35]. A 10 ms, FA =0° B_1_-independent rotation (BIR4) pre-pulse was added for the S_4_ acquisition [35]. MIX-TSE data were acquired with TSE = 8 or 16 echoes (denoted MIX-TSE-8 and MIX-TSE-16). The MIX-TSE-8 sequence was performed with TR_SE_ = 1000 ms, TR_IR_ = 2260 ms, echo time TE_1/2_ = 28.6/100 ms, inversion time TI = 0.5 s, and voxel size =0.2 × 0.2 × 2mm^3^ or 0.2 × 0.2 × 4mm^3^. MIX-TSE-16 was performed with TR_SE_ = 760 ms, TR_IR_ = 2290 ms, TE_1/2/3/4_ = 25/65/105/145 ms, TI = 0.5 s, and voxel size = 0.3 × 0.3 × 5mm^3^. The Four-FA and MIX-TSE sequences are depicted in the accompanying Additional file 1. The ‘three-point Dixon method’ [39] was used to separately image water and fat components in lesions (TR = 0.2 s; TE = 4.6, 5.8, 6.9 ms; FA = 55°; 0.27 × 0.27 × 3mm^3^ voxels).

The in vivo study utilized a 0.8 mm OD biocompatible super-elastic nitinol coaxial cable with a 42 mm whip antenna [25, 28, 29]. The coil was advanced via a femoral incision to the inferior *vena cava*, and MIX-TSE and water/fat images acquired with the identical imaging sequences used in vitro.

### Image processing

**Fig. 1:**
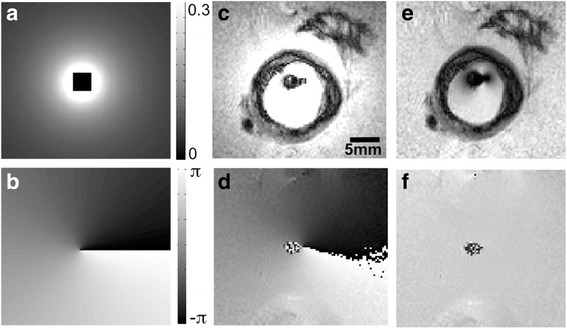
(**a**) Simulated 1/*r*-dependence of the magnitude of the IVMRI signal with radial distance from the antenna (central black square), and (**b**), the linear 0–2π phase map of the MRI signal assumed for high resolution IVMRI image correction and for SLAM reconstruction. (**c**, **e**) Magnitude and (**d**, **f**) phase 200 μm resolution MIX-TSE images of a diseased vessel from a myelodysplastic patient depicted without (**c**, **d**) and with (**e**, **f**) these corrections. The phase scale in (**b**) also applies to (**d**) and (**f**)

For the purpose of quantification, the antenna’s location was detected via its intensity/phase singularities [40] and the high resolution IVMRI signal intensity was corrected for its 1/*r* dependence with distance, *r*, from the antenna, by *r*-scaling as shown in Fig. 1, except for one case where an interpolated signal map from saline was used. The Four-FA multi-parametric maps were computed from S_1-4_ [35], and the MIX-TSE multi-parametric maps computed as prescribed [41]. The PD maps were normalized relative to a PD = 1.0 assumed for water in saline. In vitro and in vivo compartment-average T_1_, T_2_ and PD values were measured in anatomically distinct compartments of relatively uniform-appearing tissue, manually segmented from fully-sampled (*k*-space) anatomical, intensity-corrected images, and the values averaged.

### Tissue classification

A Python-based (*Python 2.7.10* interpreter, *sklearn 0.17*) linear kernel SVM classifier was trained and validated using T_1_, T_2_ and PD values from the conventional reconstructed multi-parametric maps. Because the presence of lipid within (vs. outside) vessel walls was uncommon in Dixon images in our sample set, it was excluded as an independent classifier. Data points (*n* = 248) were randomly sampled in vessel walls in artifact-free regions of the T_1_, T_2_ and PD images with adequate SNR (≥5). Healthy and diseased tissues in the freshly-harvested specimens were histologically classified as smooth muscle cells (SMC; light pink on H&E), early stage disease (green/blue on Movat, brown in Von Kossa, purple in VVG) or advanced disease (black on Movat and VVG) on sections that were anatomically co-registered with the multi-parametric maps. The samples were labeled with the histology result, and the corresponding T_1_, T_2_, and PD values. The SVM was trained using the samples and validated using the ‘leave-one-out’ [42] method. The trained SVM was also tested on the formalin-fixed specimen for robustness. The three-class SVM classifier generated probabilities for each sample belonging to each class. The class exhibiting the highest probability was designated as the output class.

A receiver operating characteristic (ROC) [43] analysis was performed to assess the potential sensitivity and specificity of IVMRI-based disease classification. For this purpose, samples were regrouped into disease and healthy SMC classes and used to train a two-class SVM classifier. The discrimination threshold was adjusted from 0 to 1 on the output disease probability to generate the ROC curve. To compare the performance of disease classification based on a single MRI parameter, the three-class and two-class SVMs were re-trained and re-tested using only one of T_1_, T_2_ or PD as the sample feature.

### SLAM IVMRI

SLAM was applied with the MIX-TSE sequence by selecting all of the high-SNR raw central *k*-space data corresponding to acceleration factors of *R* ≤ 18, i.e. by discarding up to 94% of the outer *k*-space data. Compartments that included all signal sources were segmented from the full *k*-space images of the same sections. The latter served as ‘prior knowledge’ for generating high-resolution masks for reconstructing the compartment-average signals [30, 31] (see Additional file 1). Because the magnitude and phase of the antenna’s signal exhibited strong gradients across anatomical compartments, the signal was corrected for amplitude and phase variations assuming the same 1/*r*–magnitude and linear 0–2π polar phase map depicted in Fig. 1(a, b) for the high-resolution studies. These two factors were incorporated into the SLAM reconstruction as spatially-dependent reciprocals of the receiver sensitivity profiles and phase conjugates in the ‘**A**_M*M_’ matrix in Eq. (10) of Ref. [31]. They were also factored into the discrete spatial response function (dSRF) which defines compartmental localization (see Additional file 1). T_1_, T_2_ and PD values were then computed from the compartment-average signals using the same relaxation formula used for the full *k-*space MIX-TSE MRI data [35, 41].

SLAM T_1_, T_2_ and PD values were compared with the full *k*-space results averaged over the same compartments at different acceleration factors, to test whether high-speed sensitivity-corrected SLAM could accurately characterize vessel disease components when scan-time for high-resolution relaxometry protocols is limiting.

## Results

**Fig. 2:**
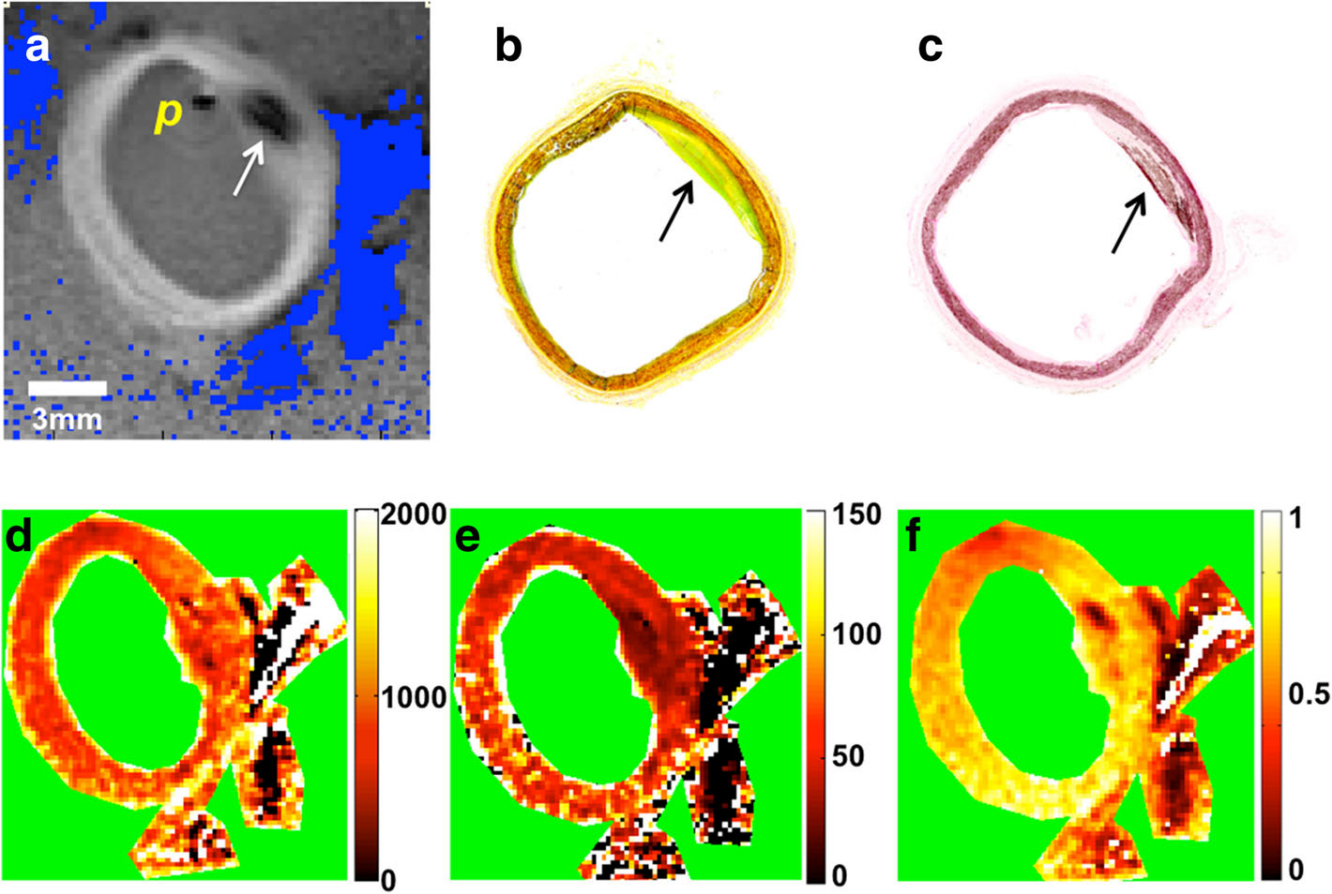
(**a**) Dixon lipid image (blue) overlaid on a gradient-echo vessel wall image, with (**b**) Movat and (**c**) Van Kossa histology results from the vessel section post-exam (arrows, calcified lesion; ‘p’ is near the probe location). Color-coded T_1_ (**d**), T_2_ (**e**), and PD (**f**) maps, below, were acquired using the Four-FA method [35]. Relaxation time scales (right) are in ms, PD is the fraction relative to water, and green denotes masking

The amplitude and phase corrections of Fig. 1(a, b) normalized the high resolution IVMRI signal intensity, as shown in a full *k*-space MIX-TSE-16 image of a vessel specimen from a patient with myelodysplastic syndrome (Fig. 1e, f). Figure 2(a) shows a 200 μm resolution IVMRI of a specimen with a plaque and peripheral fat evident in the Dixon lipid image (blue). Movat and Van Kossa staining confirm the presence of early-stage thickening and fibrosis (Fig. 2b–c). Figure 2(d–f) show the Four-FA T_1_, T_2_, and PD maps at the same resolution: the saline T_1_ and T_2_ are beyond the validation range [35] and are masked (green) for clarity.

**Fig. 3:**
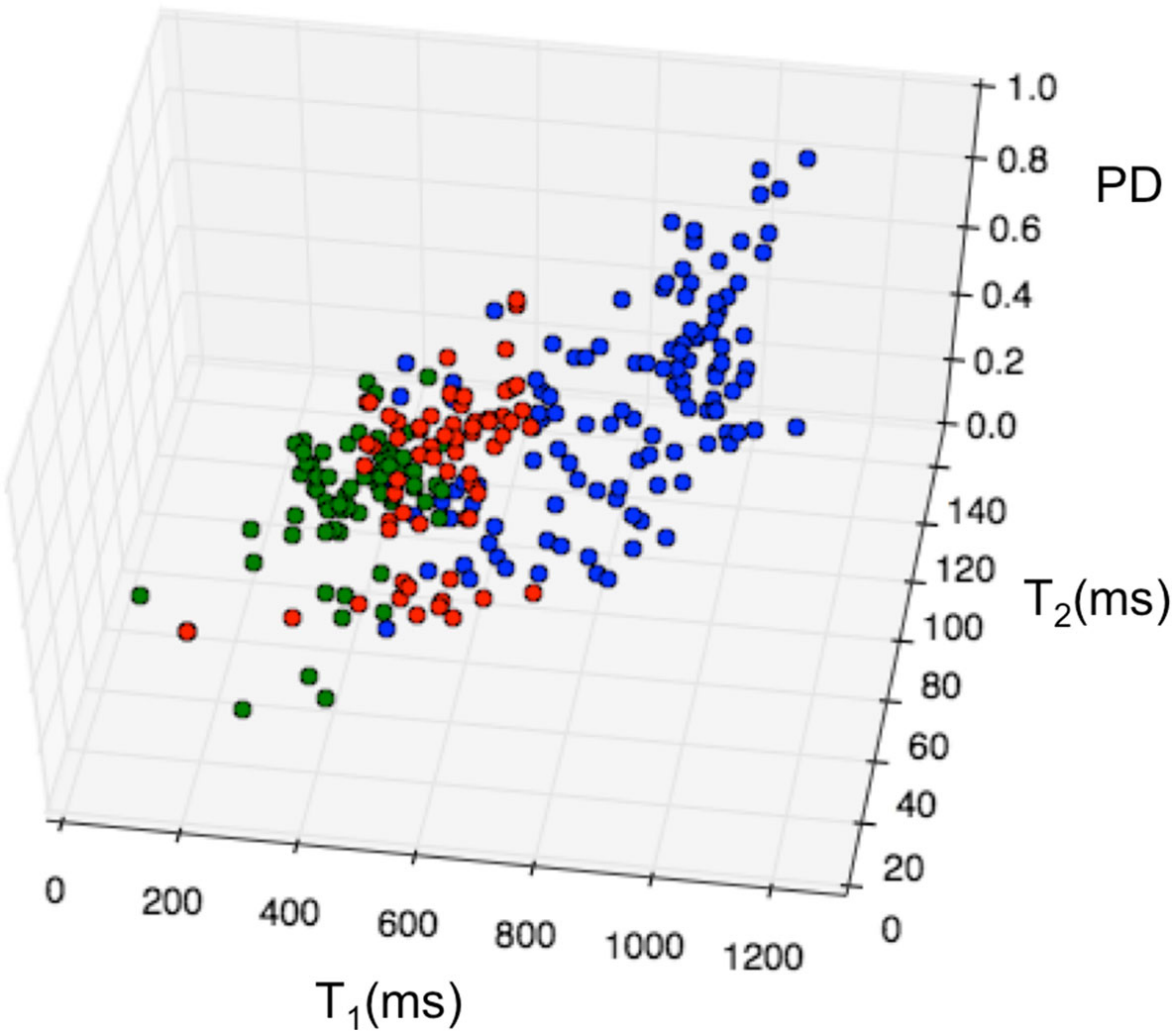
3D plot of T_1_ (ms), T_2_ (ms), and PD (relative to water) values of sampled points from three tissue classes (SMC, blue points; early disease, red; advanced disease, green)

The measured T_1_, T_2_, and PD values of the randomly sampled points from the full *k*-space images of human vessel specimens are color-coded by tissue class and plotted in Fig. 3. The means ±SD (standard deviation) summarized in Table 1, are consistent with published T_2_ values of 54 ± 13 ms [17], 76 ± 9 ms [21], 69.1 ± 6.6 ms [22] and 39 ± 5 ms [20]; and T_1_ values of 685.9 ± 166 ms [22] and 844 ± 96 ms [20], for normal vessel wall at 3 T. Published T_2_ values for lipid-rich advanced lesions are 37 ± 5 ms [17] and 54 ± 3 ms [21].

**Table 1:** Relaxometry and leave-one-out cross-validation results from the SVM automatic lesion classifier vs. histology in autopsied human aortas

	Smooth muscle	Early disease	Advanced disease
T_1_ (ms)	841 ± 155	561 ± 104	422 ± 86
T_2_ (ms)	75 ± 25	53 ± 23	51 ± 16
PD (vs. water)	0.68 ± 0.16	0.63 ± 0.17	0.54 ± 0.2
Histology classification:	116	66	66
Correctly classified by SVM:	96	43	55
Misclassified by SVM:	20	23	11

**Fig. 4:**
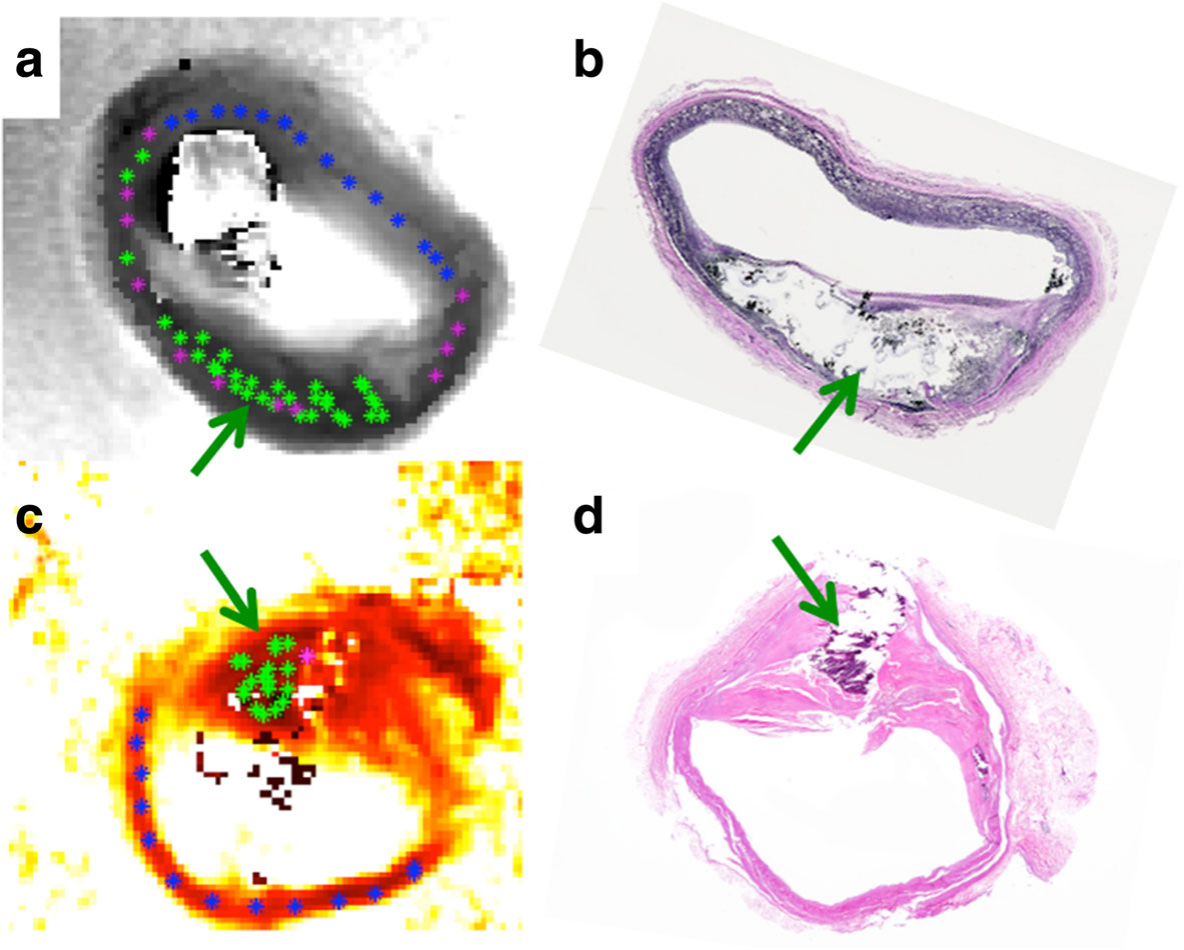
Application of the SVM classifier to random points in fresh (**a**, **b**) and formalin-fixed (**c**, **d**) vessel specimens. The points on the T_1_-weighted (**a**) and true T_1_ (**c**) images are now color-coded by the classifier as in Fig. 3 (green arrow = advanced lesion). Parts (**b**) and (**d**) are VVG-stained histological sections (some calcification is lost during staining)

The results of the ‘leave-one-out’ cross-validation of the three-class SVM are also included in Table 1. The three-class SVM correctly classified >83% of SMC and advanced lesions, and had an overall accuracy of 78%. Figure 4 exemplifies validation in a fresh vessel segment (Fig. 4a) with corresponding VVG-histology (Fig. 4b). When applied to a formalin-fixed vessel segment (Fig. 4c), the classifier correctly differentiated all advanced lesion samples (*n* = 19; mean T_1_ = 390 ± 90 ms, T_2_ = 22 ± 6 ms and PD = 0.35 ± 0.12) from SMC (*n* = 13; T_1_ = 873 ± 144 ms; T_2_ = 64 ± 11 ms; PD = 0.64 ± 0.06), as identified by histology (Fig. 4d).

**Fig. 5:**
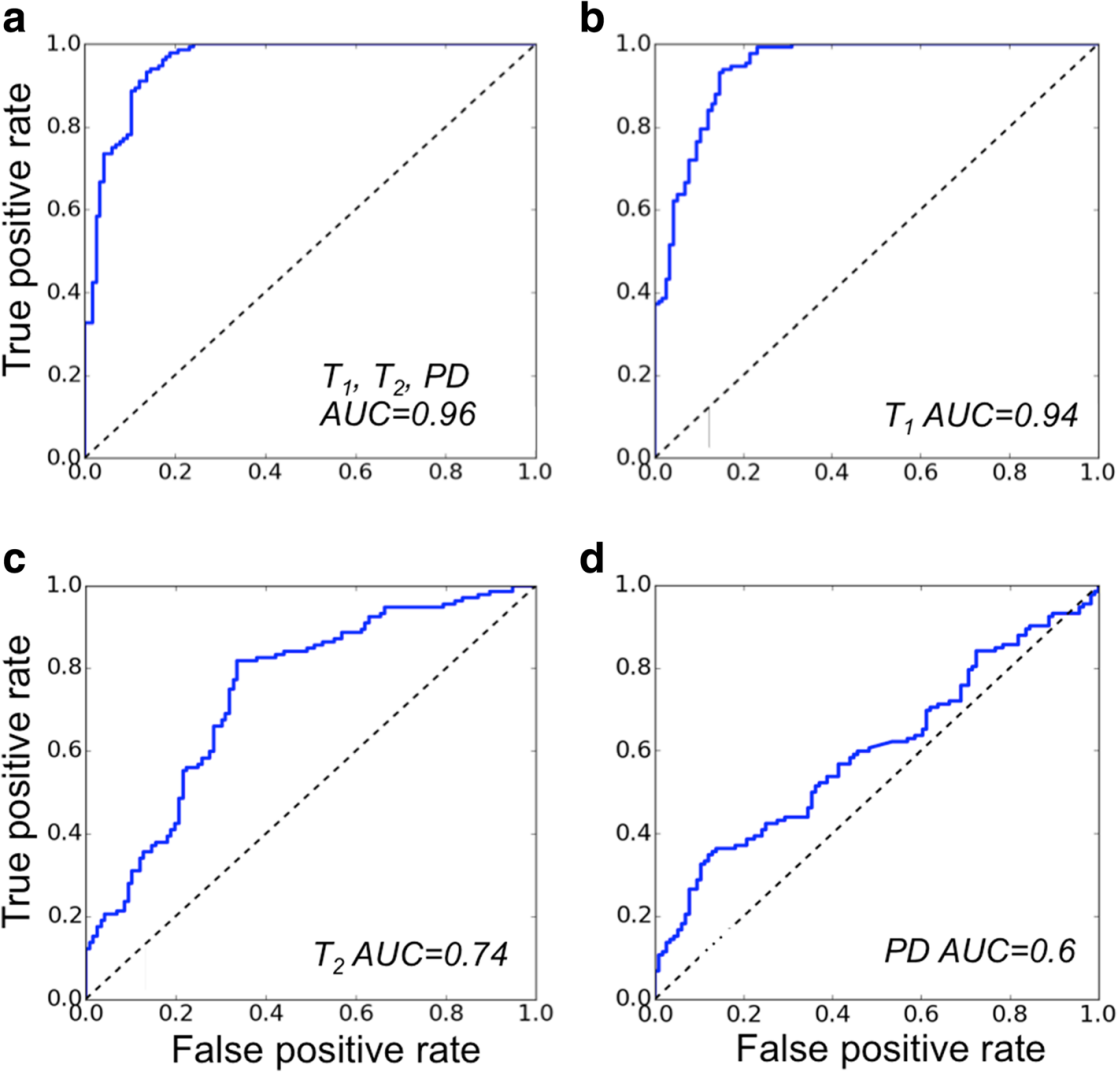
ROC curves of the classifier for distinguishing diseased and healthy tissue based on: (**a**) all three parameters, T_1_, T_2_ and PD; (**b**) T_1_ only, (**c**) T_2_, only and (**d**) PD only. Areas under the curves (AUCs) are 0.96, 0.94, 0.74 and 0.6 respectively

The two-class SVM yielded an ROC curve for distinguishing disease from non-disease with the highest overall accuracy and area under curve (AUC) of 0.96 using all three parameters (Fig. 5a). With the three parameters tested separately, T_1_ afforded the highest sensitivity and specificity for differentiating vessel disease from healthy tissue, with an AUC = 0.94, that accounted for nearly all of the three parameter result (Fig. 5b–d). By itself, PD was least able to discriminate disease (AUC = 0.6). The two-class SVM correctly classified the SLAM data as well, including *vena cava,* in vivo.

**Fig. 6:**
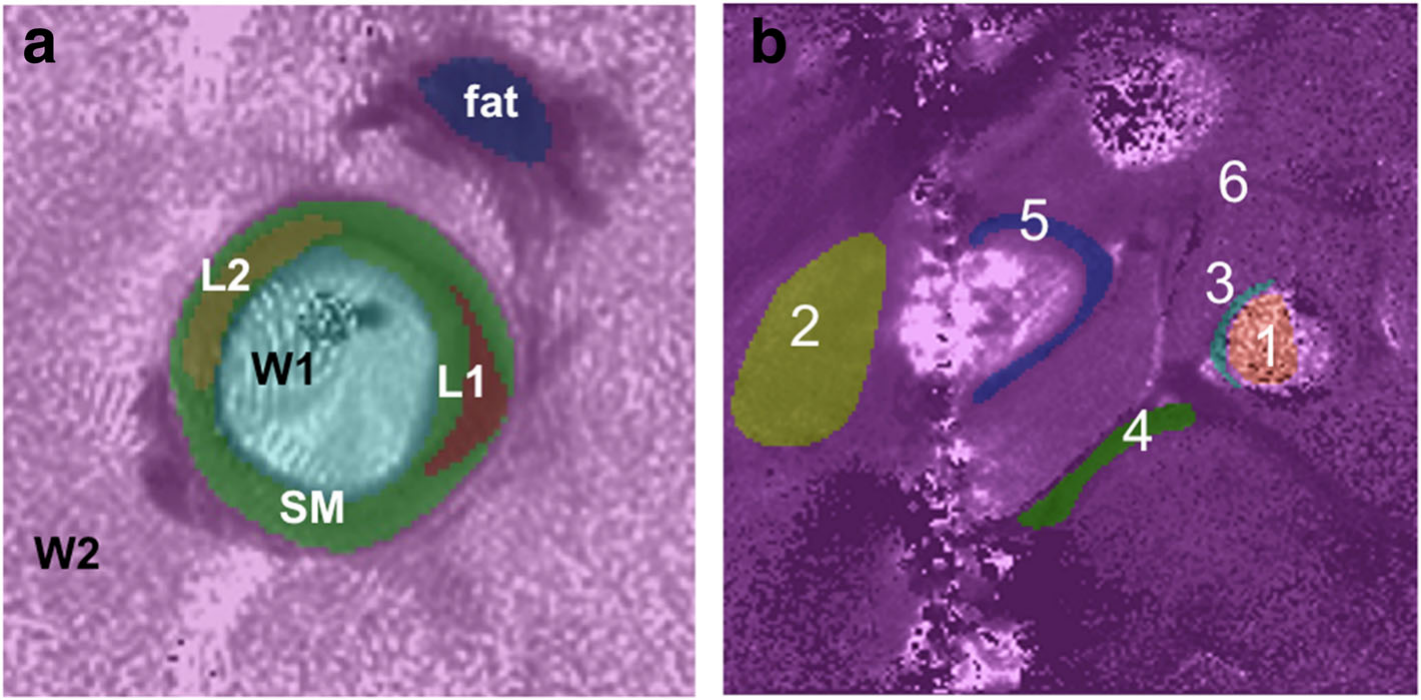
(**a**) Six anatomical compartments segmented for SLAM reconstruction overlaid on a T_1_-weighted IV image from a diseased vessel specimen (fat; two lesions, L1 and L2; vessel fluid contents, W1; smooth vessel-wall muscle, SM; surrounding tissue = W2). (**b**) In vivo SLAM segmentation of six compartments on an in vivo T_1_ image of the inferior *vena cava* (1, blood; 2, surrounding tissue; 3, arterial wall; 4, fat stripe; 5, vein wall; 6, everything else)

Applying the amplitude and phase corrections from Fig. 1(a, b) to SLAM T_1_, T_2_ and PD measurements with *R* = 10-fold acceleration (effective scan time = 14.3 s for all three parameters) dramatically improved the accuracy of the larger vessel compartments in Fig. 6(a) (W2, SM, Fat–PD), as compared to no correction (Table 2). These compartments encompass extended portions of the antenna’s inhomogeneous sensitivity profile, while in the two smaller lesion compartments (L1, L2), the sensitivity variations are insufficient to corrupt the mean measurements provided by SLAM without the homogeneity corrections. The elongated dSRF of compartment L1 is plotted in Additional file 1.

**Table 2:** Effect of magnitude and phase corrections on SLAM relaxation and PD measurements with R = 10-fold acceleration

	No correction	With Correction	True value^a^
W2 T_1_ (ms)	3700	2780	2700 ± 800
W2 T_2_ (ms)	−15,240	1320	1200 ± 1380
SM PD (vs. water)	0.2	0.83	0.82 ± 0.2
Fat T_1_ (ms)	370	373	370 ± 100
Fat T_2_ (ms)	104	104	112 ± 31
Fat PD (vs. water)	0.44	0.7	0.67 ± 0.11
L1 T_1_ (ms)	493.4	507	514 ± 98
L1 T_2_ (ms)	52	51.7	53.4 ± 7.5
L1 PD (vs. water)	0.89	0.89	0.84 ± 0.09
L2 T_1_ (ms)	509.5	490.5	475 ± 127
L2 T_2_ (ms)	70	69	67.2 ± 16
L2 PD (vs. water)	0.67	0.63	0.64 ± 0.12

^a^Mean ± SD for each compartment defined in Fig. 6(a) as measured from the full *k*-space data sets. SM = smooth vessel-wall muscle; W2 = surrounding tissue; L1 = lesion 1; L2 = lesion 2

**Fig. 7:**
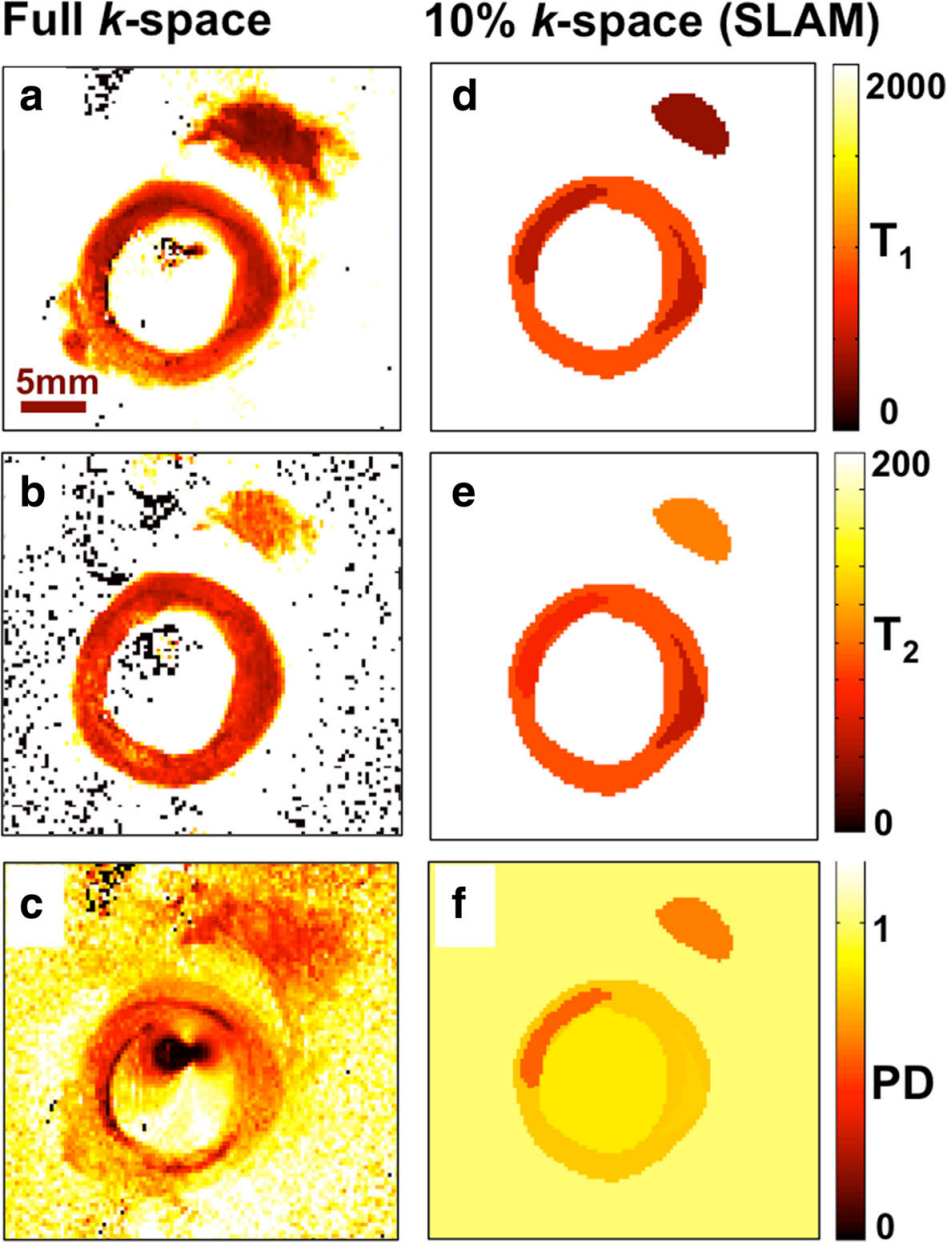
Color-coded T_1_ (ms; **a**, **d**), T_2_ (ms; **b**, **e**) and PD (=1 for water; **c**, **f**) maps calculated using the full *k*-space MIX-TSE data (**a**–**c**); and with SLAM using only 10% of the central *k*-space data (**d**–**f**) and the magnitude and phase corrections shown in Fig. 1(a–b)

**Fig. 8:**
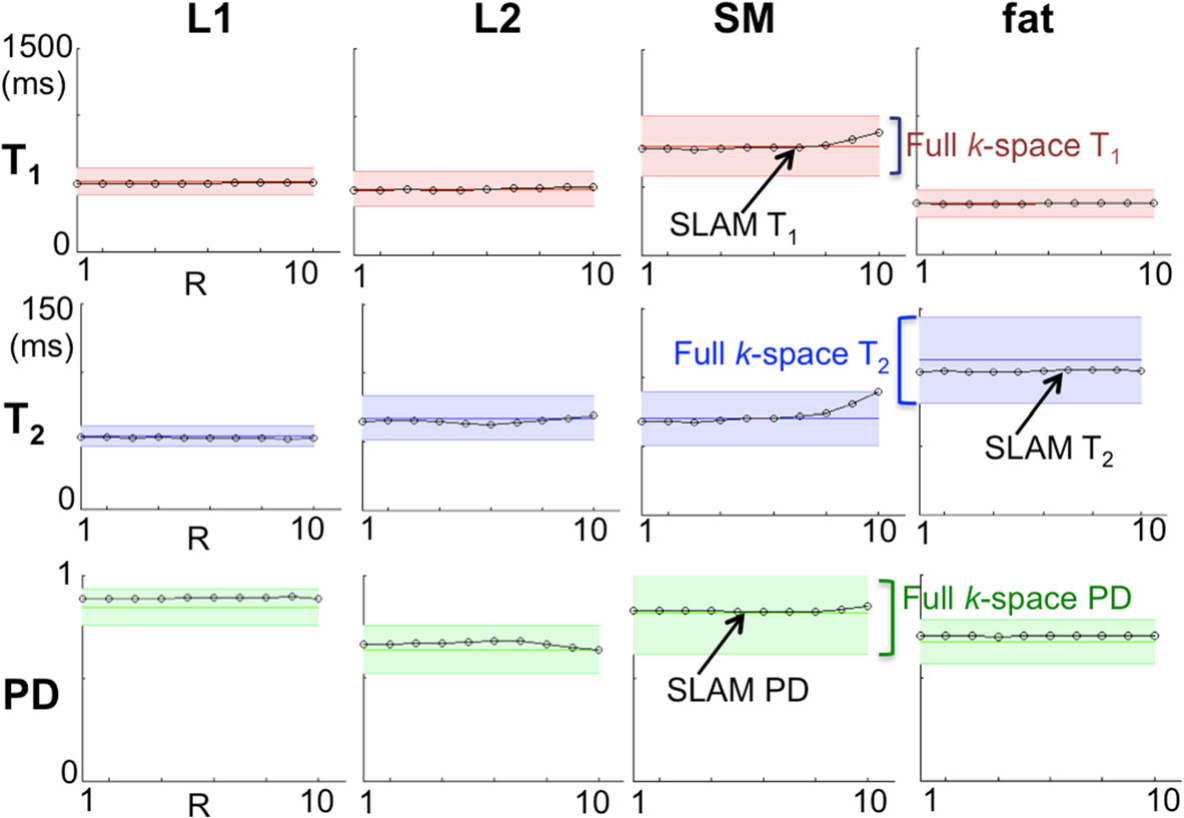
SLAM T_1_, T_2_ and PD values from four compartments of the autopsy specimen shown in Fig. 3(a) as a function of *R*. Colored error bands denote compartment mean values ±SD as derived from the corresponding full *k-*space images. SLAM and full *k-*space measures of T_1_, T_2_ and PD in fluid and surrounding tissue compartments (not shown) also agree

Post-correction, the SLAM T_1_, T_2_ and PD parametric maps are consistent with the regular (full *k*-space) MIX images (Fig. 7): the ranges are plotted as a function of *R* in Fig. 8. For *R* ≤ 10, SLAM T_1_, T_2_ and PD measurements fall within a standard deviation (SD) for all compartments. The mean values of all parameters in lesion and fat compartments differ from full *k-*space measures by ≤0.5%(±4%SD). Even in surrounding tissue and fluid within the vessel where the SDs of the means of T_1_ and PD from the full *k-*space images are ≥30% (due to low SNR), SLAM T_1_ and PD values agreed with the means to within ≤6%(±6%SD; data not shown).

In vivo, SLAM reconstruction of six compartments (Fig. 6b; 1, blood; 2, surrounding tissue; 3, artery wall; 4, fat stripe; 5, vein vessel wall; 6, ‘everything else’) also yielded T_1_, T_2_ and PD values that fell within a SD of the mean compartment values measured from the full *k*-space data, at least up to *R* = 18 (Table 3). Note that compartment 6 (‘everything else’) is a catch-all residual created to meet the criterion that all signal sources be allocated for SLAM reconstruction [31]: it is not meaningful and has a huge SD.

**Table 3:** Comparison of in vivo vessel compartment-average T_1_ (ms), T_2_ (ms), and PD (relative to water) measured using SLAM with acceleration factors of R = 1-18, and those measured using the full *k*-space reconstruction

	Comp.^a^	R = 1	R = 3	R = 6	R = 10	R = 18	full *k-*space^b^
T_1_	1	2916	2905	2900	2946	2826	2802 ± 721
	2	787	787	788	786	780	812 ± 124
	3	848	865	993	1094	1041	929 ± 388
	4	236	237	243	288	365	253 ± 122
	5	731	728	740	804	879	776 ± 123
	6	705	705	704	705	704	964 ± 704
T_2_	1	1173	1154	1131	1040	825	1050 ± 1253
	2	47	47	47	47	47	51 ± 15
	3	75	81	106	123	135	128 ± 247
	4	99	99	98	93	86	98 ± 21.5
	5	42	42	42	43	47	46 ± 10
	6	64	64	64	63	64	101 ± 176
PD	1	1.01	1.01	1.00	1.00	0.95	1.00 ± 0.24
	2	0.77	0.77	0.78	0.78	0.77	0.74 ± 0.13
	3	0.45	0.44	0.45	0.50	0.52	0.46 ± 0.18
	4	0.41	0.40	0.40	0.39	0.38	0.45 ± 0.10
	5	0.81	0.81	0.82	0.84	0.83	0.84 ± 0.18
	6	0.40	0.40	0.40	0.40	0.40	0.65 ± 0.54

^a^Compartments as defined in Fig. 6(b)

^b^ Mean ± SD of compartment

## Discussion

The purpose of this study was two-fold: first, to test whether IVMRI measurements of T_1_, T_2_, PD, and lipid imaging could be used to characterize vessel disease in a clinical 3 T scanner; and second, to develop a strategy that could accelerate quantification of these metrics and enable disease characterization in situations where multi-parametric acquisitions are limited by scan-time. To date, the classification of vessel disease into stages I-VI using the AHA scale [2] with minimally-invasive X-ray, IVUS and OCT imaging is confounded by poor soft-tissue sensitivity, artifacts and/or the modality’s limited depth penetration [3–6]. The characterization of vessel-wall pathology using conventional MRI, which *is* intrinsically sensitive to soft-tissue, is limited by spatial resolution and long scan-times for T_1_, T_2_, and PD measurements.

Here, spatial resolution is addressed using IVMRI detectors that offer locally-intense SNR with ~200 μm resolution at ≥3 T [25–27, 29]. Such resolution is not currently achievable with external detectors in clinical scanners. Together with the high vessel-wall SNR afforded by IVMRI, high resolution can avoid the confounding partial-volume effects and signal ‘bleed’ from adjacent flowing blood. We used a machine-learning SVM classifier trained on histology to automatically differentiate three classes of healthy and atherosclerotic tissue and test the suitability of T_1_, T_2_ and PD for classifying vessel disease. The automatic classifier demonstrated an accuracy of ~80%, with an AUC of 0.96 in ROC analysis for disease detection overall (Fig. 5).

Few studies have reported multi-parametric mapping in vessel walls using clinical scanners at 3 T. The use of relaxation-based ‘black blood’ MRI methods to eliminate contaminating blood signals (e.g. using inversion or saturation) may be a contributing factor as these can confound T_1_ relaxometry, including that performed with the minimum-acquisition multi-parametric sequences deployed here. Indeed, most vessel wall studies have focused on T_2_ mapping [17, 21] which was believed to better highlight disease [21, 44]. However, except for formalin-fixed tissue, we found that T_1_ better-discriminated disease from healthy tissue than T_2_ and PD alone (Fig. 5), while T_1_ provided a comparable contrast to T_2_ for distinguishing early and advanced lesions (Fig. 3, Table 1). In formalin-fixed specimens, the shorter T_2_ (22 ms in main text vs 51 ms in Table 1) and lower PD (0.35 vs 0.54 in Table 1) combined with the T_1_ differences to improve the accuracy of the automatic classifier to near 100% for distinguishing advanced disease, without any retraining. High T_1_–contrast was noted for vessel wall components measured at 9.4 T in the only other report we found with both T_1_ and T_2_ data from atherosclerotic tissue [45]. However, as evident from the published values listed in ‘Results’, prior vascular relaxation measurements lack consensus, which may reflect the spatial resolution, SNR, and scan-time limitations of conventional approaches. Even so, our measured T_1_ and T_2_ values lie within the range of previous results [17, 19–22].

To our knowledge, this study is the first to combine multi-parametric MRI mapping with an advanced machine learning algorithm to classify plaques. However, plaque characterization using only T_2_ and a fuzzy ‘C-means’ classifier has been reported [46]. We found that the combination of all three parameters, T_1_, T_2_ and PD, provided the most accurate disease classification in 3D ‘feature space’ (Table 1, Figs. 3 and 5). The SVM approach maps the parametric data to a high-dimensional space to compute a hyper-plane that optimizes the separation of the training-dataset into the designated classes. The three-class SVM classifier was robust to varying sample conditions as evidenced by its ability to correctly classify tissue in refrigerated, formalin-fixed and fresh specimens (Fig. 4), as well as healthy in vivo tissue after training on fresh specimens alone. The SVM partitions the 3D space with decision planes such that the vectors normal to the planes are in directions that maximally separate data points from the two adjacent classes. Therefore, weighted images acquired with sequence parameters that correspond to the normal direction of the decision plane should on average provide the maximum contrast between two adjacent classes.

A prior study at 1.5 T reported that qualitative assessments of lesion appearance in time-of-flight, T_1_- and T_2_-weighted MRI could be used for AHA disease classification [14]. Our studies indicate that T_1_, T_2_, and PD may suffice to discriminate atherosclerotic lesion stage, and that a three-parameter SVM classifier could do so automatically. The SVM might identify additional AHA classifiers or features [2] as well, but the present study was constrained by the availability of fresh human vessel specimens exhibiting a sufficient number of histologically diverse pathologies needed for training. Note that training the SVM classifier is cumulative: adding more data from further studies will better tune its accuracy and define its potential for quantitative IVMRI disease classification. Although our data sampled specimens from a limited group of decedents, the training of machine-learning classifiers does not require independent training samples [47], and the versatility of our trained SVM classifier was demonstrated by its success with formalin-fixed specimens. With enough pathology samples a plaque component classifier might ultimately be able to automatically identify calcifications (stage V), which appear as signal voids in scout and endoscopic images [25]; fibrous caps (stages II-III) which are measurable by IVMRI [29]; lipids in AHA class I-III lesions revealed by Dixon imaging (Fig. 2a); and perhaps even vulnerable plaque to the extent it is identifiable in training specimens and distinguishable via its IVMRI parameter set.

Our strategy to speed-up the acquisition of multi-parametric T_1_, T_2_, and PD mapping by employing sensitivity-weighted SLAM reconstruction [30, 31], yielded compartment-average measurements up to *R* = 18 times faster than regular IVMRI. This makes an extraordinary dent on the long scan-times conventionally required for parametric mapping, and reduces opportunities for physiological motion to corrupt data in vivo. A virtue of SLAM is that the compartments which are segmented from high-resolution scout scans, can be re-adjusted at will from the same data set post-acquisition [34]. In principle, the acceleration factor, *R*, for SLAM reconstruction can be increased by reducing the number of phase-encoding steps until it equals the number of compartments: the maximum is *R* = 30 for Fig. 6, for example. By encoding compartments directly using the highest-SNR spatial encoding steps, SLAM realizes an SNR gain roughly proportional to the square-root of the number of voxels in the compartment, as compared to summing the same voxel signals from the high-resolution image, post-acquisition [30–34]. This partially offsets the SNR lost by reducing the acquisition time. In contrast, the application of compressed-SENSE undersampling to IVMRI at 3 T using the same nominal spatial resolution, realizes no volume SNR gain that could offset the SNR loss from acceleration, and acceleration has been limited to *R* ≤ 4 [40].

SLAM assumes a uniform signal within each compartment wherein the reconstruction is an exact solution of a set of simultaneous equations (see Additional file 1) [30, 31]. While as few as one voxel can be assigned to a compartment from the segmentation image, an SNR penalty proportional to the compartment volume is paid as compartments are shrunk. Deviations from the uniformity assumption can introduce bleed error [30], which is ameliorated using the ‘SLAM2’ algorithm [30, 31, 34]. Here, simple linear 0–2π phase and *r*-corrections adequately addressed the loopless antenna’s highly nonuniform sensitivity profile, as evidenced by SLAM’s accuracy up to *R* = 10 (Fig. 8). Owing to the device’s cylindrical symmetry [27], the correction is relatively robust to antenna orientations askew from the B_0_-axis, although such corrections may not suffice for larger *R* values, especially for larger compartments encompassing larger areas of nonuniformity. Nevertheless, SLAM offers dramatic reductions in scan-times allowing quantitative multi-parametric IVMRI in ≤15 s, even in regions of low SNR.

## Conclusion

High-resolution, sensitivity-corrected, multi-parametric IVMRI in combination with a supervised machine learning SVM classifier can automatically classify normal, early and advanced atherosclerosis with ~80% accuracy and an AUC of ~0.96 based on T_1_, T_2_ and PD measurements. Replacing IVMRI relaxometry with sensitivity-corrected intravascular SLAM relaxometry provides equivalent estimates of T_1_, T_2_ and PD an order-of-magnitude faster to facilitate IVMRI-based characterization of vessel disease.

A possible *modus operandi* for this technology is to perform high-resolution 3 T scout IVMRI using a regular gradient-echo or steady-state free-precession sequence with MRI endoscopy at 1-2 fr/s [25], or another high-speed undersampling method [40]. At a region of suspected disease, the sequence would be switched to a T_1_/T_2_ acquisition employing a Four-FA [35], MIX-TSE or other high-speed relaxometry method; but with all of the phase-encode steps omitted save for, say, 6-10 steps lying at the center of image *k*-space to provide an order(s)-of-magnitude speed-up. Post-acquisition, 6-10 compartments of interest would be segmented from the regular IVMRI, and their T_1_, T_2_ and PD values reconstructed using the sensitivity-corrected SLAM algorithm presented herein. Vessel disease could then be characterized automatically with the SVM classifier.

## Additional file

Additional file 1: Supplementary file. (PDF 2140 kb)

## Abbreviations

2D: Two-dimensional
3D: Three-dimensional
AHA: American Heart Association
AUC: Area under curve
BIR4: B_1_-independent rotation
FA: flip-angle
Four-FA: Four flip-angle
H&E: Hematoxylin and eosin
IV: Intravascular
IVUS: Intravascular ultrasound
MIX-TSE: Mixed turbo spin-echo
MRI: Magnetic resonance imaging
OCT: Optical coherence tomography
OD: Outer diameter
PD: Proton density
ROC: Receiver operating characteristic
SD: Standard deviation
SENSE: Sensitivity encoding
SLAM: Spectroscopy with linear algebraic modeling
SMC: Smooth muscle cells
SNR: Signal-to-noise ratio
SVM: Support vector machine
T_1_: Spin-lattice relaxation time
T_2_: Spin-spin relaxation time
TE: Echo time
TI: inversion time
TR: sequence repetition period
VVG: Verhoeff-van Gieson
