# Supplementary material for: High-resolution and accelerated multi-parametric mapping with automated characterization of vessel disease using intravascular MRI
Source: J Cardiovasc Magn Reson. 2017 Nov 20;19:89. doi: 10.1186/s12968-017-0399-6 (PMC5694914; doi:10.1186/s12968-017-0399-6)
Supplement: Additional file 1: — Supplementary file. (PDF 2140 kb) [file 12968_2017_399_MOESM1_ESM.pdf]

Supplement: "High-resolution and accelerated multi-parametric mapping with automated characterization of vessel disease using intravascular MRI"

MRI sequences

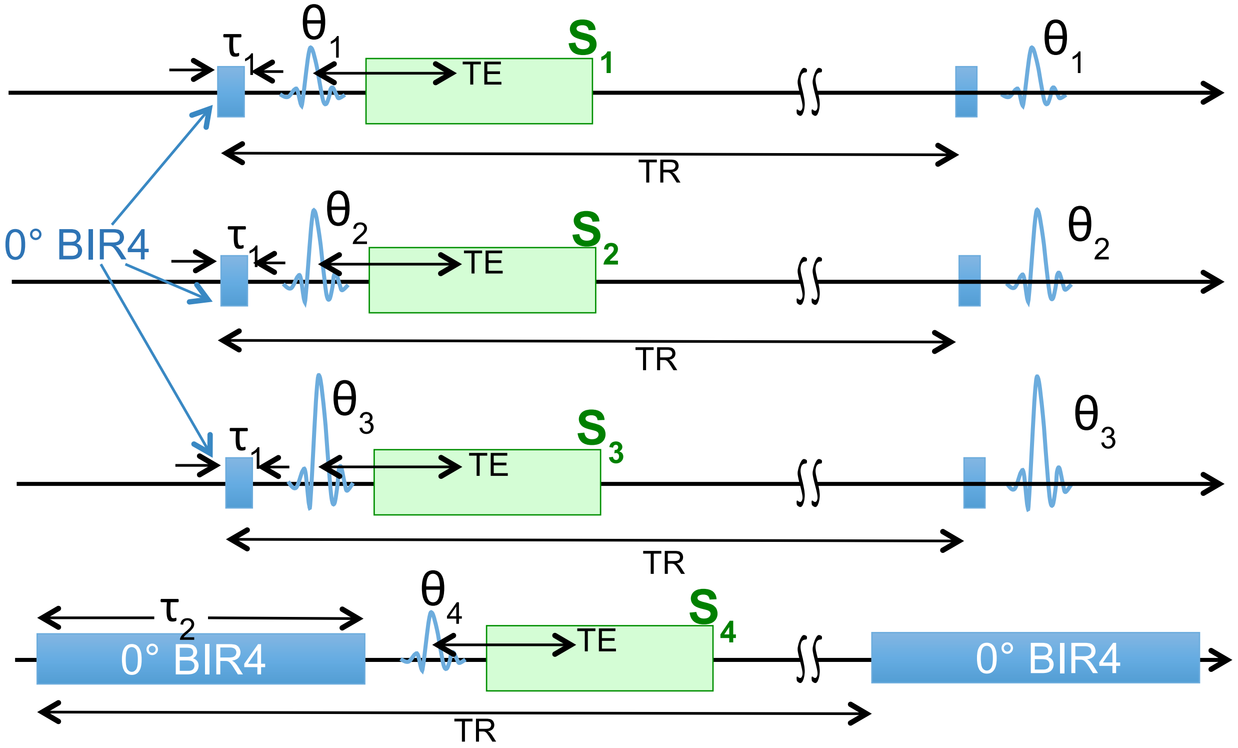

Fig. S1: Timing diagram for the Four-FA method of measuring and imaging T_1, T_2 and PD [35]. Four gradient echo MRI sequences are applied at steady-state equilibrium to generate 4 image sets, S_1-S_4 using different nominal excitation flip-angles of $\theta_{1\text{-}4}$ (30°, 80°, 140°, 30°). The sequences are preceded by 0° adiabatic B_1-independent rotation (BIR4) pulses of short ($\tau_1 << T_2$; S_1-S_3) or long duration ($\tau_2 \sim T_2$; S_4). The same sequence repetition period, TR, was used for these studies. The Four-FA method incorporates corrections for RF field (B_1) nonuniformity: T1 and B1 are measured and imaged by the variable flip-angle method, and T2 is encoded via the signal decay during τ_2. The T_1, T_2 and PD information is extracted by least squares fitting of the four signal equations [35].

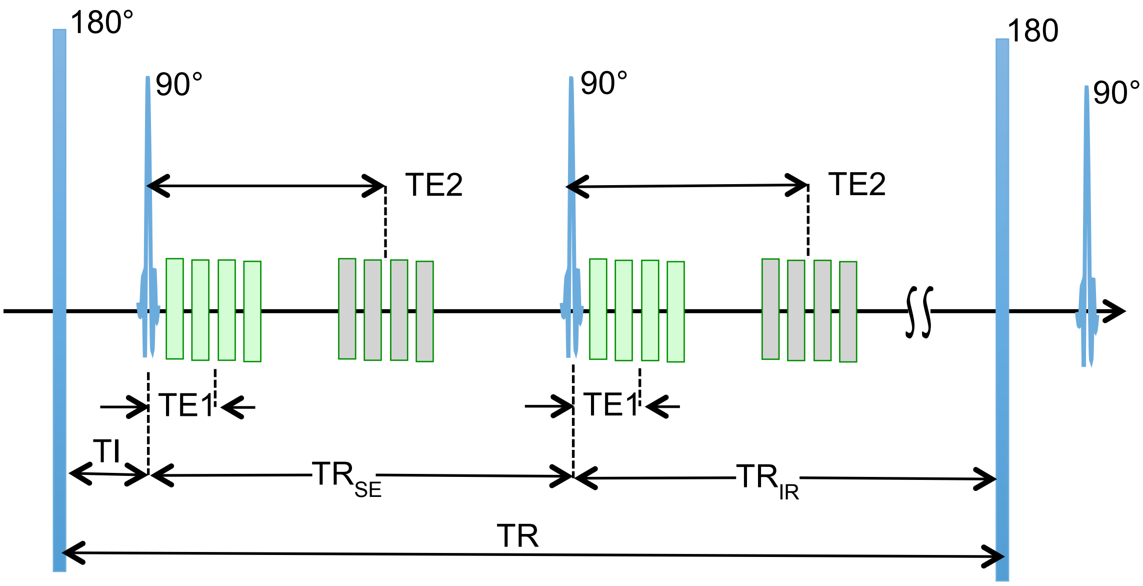

Fig. S2: Timing diagram for a mixed turbo spin-echo (MIX-TSE-8) MRI sequence. An inversion recovery is performed with inversion time TI followed by a 90° readout pulse and gradients to stimulate two sets of echoes at times TE1 and TE2. Multiple lines of image *k*-space are readout during each echo set to generate data acquired at 2 different echo times. A second 90°excitation pulse is applied at time TR_{SE} after the first, another pair of echoes stimulated, and more lines of *k*-space read before the whole sequence is repeated at time TR_{IR}. T_1, T_2 and PD are calculated using a standard algorithm on the Philips scanner ([36] and Refs. therein)

SLAM reconstruction

The standard discrete Fourier Transform reconstruction model can be cast as:

$$\mathbf{s}_{M'} = \mathbf{E}_{M'*M} \times \boldsymbol{\rho}_M \tag{1}$$

where **s** is the raw *k*-space signal vector, **E** is the combined phase and frequency encoding matrix; **ρ** is the image space data; *M* is the number of image-space voxels; *M'* is the total number of known *k*-space data equal to the product of the number of phase-encoding steps and number of frequency-encoding steps; " * " denotes adjoining matrix dimensions; and " × " denotes matrix multiplication. For full *k*-space sampling, *M'*=*M*.

In a SLAM experiment segmentation information from *C* anatomical compartments is incorporated into an auxiliary **b** matrix described previously [31, 33], while the reciprocals of the complex IVMRI receiver sensitivity profile and receiver phase conjugates are folded into a

matrix, $\mathbf{A}_{M*M}$, according to Eqn. (10) of Ref. [31]. Denoting their inverses as $\mathbf{b}^{-1}$ and $\mathbf{A}^{-1}$, Eq. (1) can be rewritten as:

$$\mathbf{s}_{M'} = \mathbf{E}_{M'*M} \times \mathbf{A}_{M*M}^{-1} \times \mathbf{b}_{M*M}^{-1} \times \mathbf{b}_{M*M} \times \mathbf{A}_{M*M} \times \boldsymbol{\rho}_M . \tag{2}$$

The $\mathbf{b}$ matrix is constructed by inserting "-1" elements into an identity matrix in order to zero out redundant rows in $\boldsymbol{\rho}$ that belong to the same compartment.

As a simple example, suppose there are total 8 voxels in image space, wherein voxels #1-3 are in compartment 1, and voxels #4-8 are in compartment 2. The corresponding $\mathbf{b}$ matrix is formed as:

$$\mathbf{b} = \begin{bmatrix} 1 & 0 & 0 & 0 & 0 & 0 & 0 & 0 \\ -1 & 1 & 0 & 0 & 0 & 0 & 0 & 0 \\ -1 & 0 & 1 & 0 & 0 & 0 & 0 & 0 \\ 0 & 0 & 0 & 1 & 0 & 0 & 0 & 0 \\ 0 & 0 & 0 & -1 & 1 & 0 & 0 & 0 \\ 0 & 0 & 0 & -1 & 0 & 1 & 0 & 0 \\ 0 & 0 & 0 & -1 & 0 & 0 & 1 & 0 \\ 0 & 0 & 0 & -1 & 0 & 0 & 0 & 1 \end{bmatrix}. \tag{3}$$

Thus, only rows #1 and 4 in $\boldsymbol{\rho}$ are intended to be kept. The sensitivity/phase corrected $\mathbf{A}$ matrix is constructed as:

$$\mathbf{A} = \begin{bmatrix} a_1 & 0 & 0 & 0 & 0 & 0 & 0 & 0 \\ 0 & a_2 & 0 & 0 & 0 & 0 & 0 & 0 \\ 0 & 0 & a_3 & 0 & 0 & 0 & 0 & 0 \\ 0 & 0 & 0 & a_4 & 0 & 0 & 0 & 0 \\ 0 & 0 & 0 & 0 & a_5 & 0 & 0 & 0 \\ 0 & 0 & 0 & 0 & 0 & a_6 & 0 & 0 \\ 0 & 0 & 0 & 0 & 0 & 0 & a_7 & 0 \\ 0 & 0 & 0 & 0 & 0 & 0 & 0 & a_8 \end{bmatrix}, \tag{4}$$

where $a_l = \sqrt{(x_l - x_0)^2 + (y_l - y_0)^2} \cdot \exp(i \cdot \arctan(\frac{y_l - y_0}{x_l - x_0}))$, (x_l, y_l) is the discretized spatial location of the lth voxel ($l = 1...8$) and (x_0, y_0) is the spatial location of the IVMRI antenna.

After selecting columns corresponding to the first voxel index of each compartment in $\mathbf{b}^{-1}$ (e.g. #1 and #4 as in the example), Eq. (2) can be reduced to

$$\mathbf{s}_{M'} = \mathbf{E}_{M'*M} \times \mathbf{A}^{-1}_{M*M} \times \mathbf{b}'^{r}_{M*C} \times \boldsymbol{\rho}'^{r}_{C}, \quad (5)$$

where $\mathbf{b}^{r}$ preserves the C columns in $\mathbf{b}^{-1}$ corresponding to the C compartmental average values in $\boldsymbol{\rho}^{r}$, and $\boldsymbol{\rho}'^{r}_{C}$ is a submatrix of the C non-eliminated rows of $\mathbf{b}_{M*M} \times \mathbf{A}_{M*M} \times \boldsymbol{\rho}_{M}$. The compartmental signals, $\boldsymbol{\rho}'^{r}_{C}$, are reconstructed by solving:

$$\boldsymbol{\rho}'^{r}_{C} = (\mathbf{E}_{M'*M} \times \mathbf{A}^{-1}_{M*M} \times \mathbf{b}'^{r}_{M*C})^{+} \times \mathbf{s}_{M'} \quad (6)$$

$$\text{or } \boldsymbol{\rho}'^{r}_{C} = (\mathbf{b}'^{r}_{M*C})^{+} \times \mathbf{A}_{M*M} \times (\mathbf{E}_{M'*M})^{+} \times \mathbf{s}_{M'} \quad (7)$$

where "+" denotes the Moore-Penrose pseudo-inverse. The two reconstructions are denoted SLAM1 and SLAM2, respectively [31, 34]. SLAM2, which is more robust to intra-compartmental signal heterogeneity, was used in the present study.

Discrete spatial response function (dSRF) for SLAM

The spatial resolution of a compartment segmented by SLAM can be characterized by the dSRF which depends on the distribution and size of the compartments, and the acceleration factor [31, 33, 34]. With the sensitivity and phase corrections included,

$$\mathbf{dSRF}_{C*M} = (\mathbf{E}_{M'*M} \times \mathbf{A}^{-1}_{M*M} \times \mathbf{b}'^{r}_{M*C})^{+} \times \mathbf{E}_{M'*M} \times \mathbf{A}^{-1}_{M*M} \quad (8)$$

$$\text{and } \mathbf{dSRF}_{C*M} = (\mathbf{b}'^{r}_{M*C})^{+} \times \mathbf{A}_{M*M} \times (\mathbf{E}_{M'*M})^{+} \times \mathbf{E}_{M'*M} \times \mathbf{A}^{-1}_{M*M} \quad (9)$$

for SLAM1 and SLAM2 respectively. The observed compartment signal is equal to the integral of the product of the dSRF with the sensitivity and phase-corrected signal distribution, over the segmented compartment [28]. The leakage or error signal, is equal to the integral of the product of the dSRF with the corrected signal distribution, over the volume outside of the segmented compartment [31]. When the signal distribution is uniform within compartments, the spatial fluctuations in dSRF for SLAM1 cancel upon integration to satisfy Eq. (6).

As an example, the sensitivity-corrected dSRF for the smallest lesion, L1 in Fig. 6a, is plotted in Fig.S3 as a function of the acceleration factor $R \leq 18$. The uniformity of the dSRF deteriorates as R increases, but because the observed signal is the integral of the dSRF with the signal distribution, accuracy is fairly well-preserved up to $R=10$ (Fig. 8, Table 2).

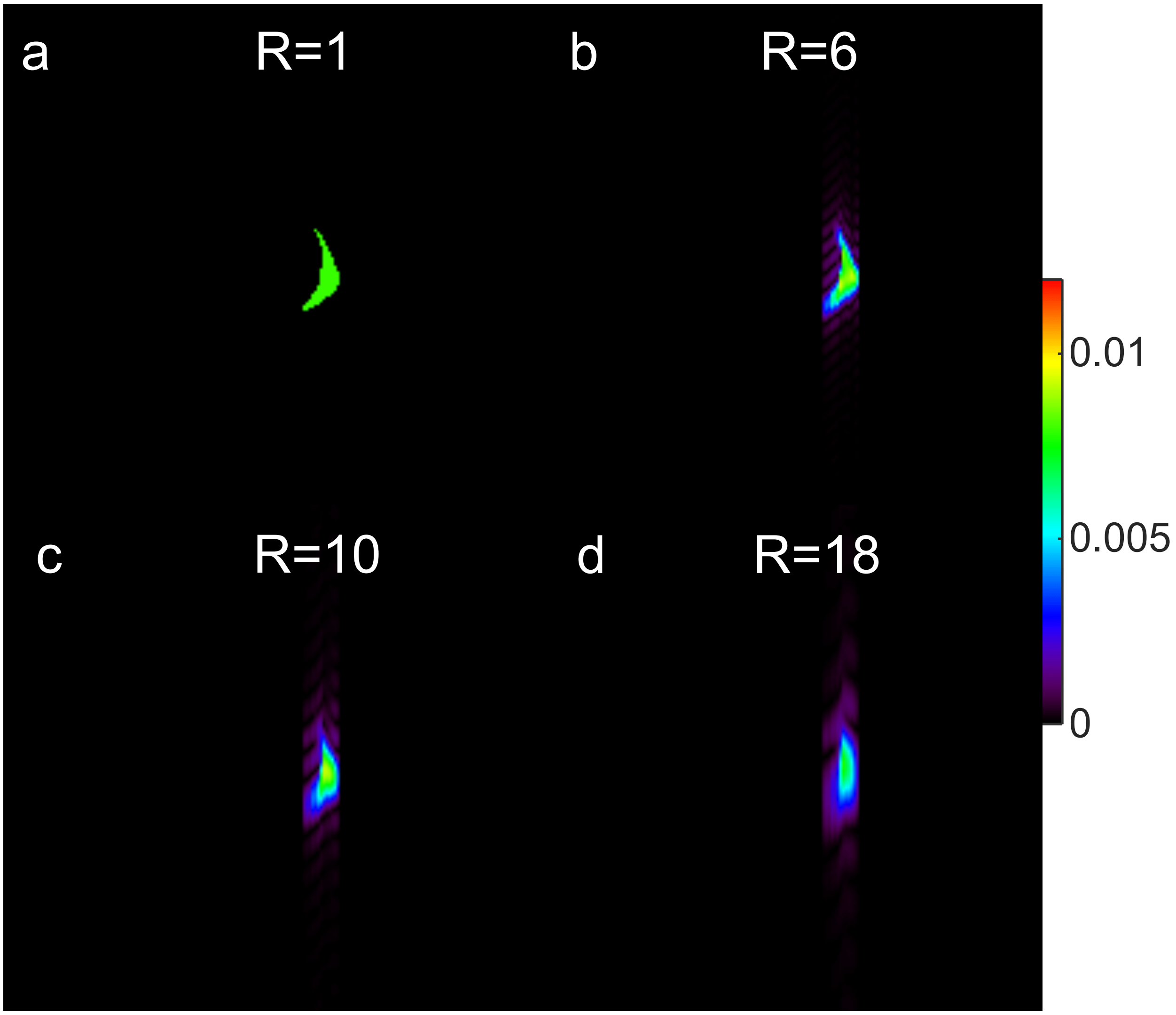

Fig. S3: SLAM2 dSRF for the smallest lesion, L1 in Fig. 6a, plotted for acceleration factors of (a) R=1 (no acceleration), (b) R=6, (c) R=10 and (d) R=18. This compartment occupies 126 voxels out of a total of 33,120 voxels in the segmentation image. The color bar is from 0 to 0.012 such that the integral of the dSRF across the whole field of view is 1.0.
